# Oncologic and long-term outcomes of enhanced recovery after surgery in cancer surgeries — a systematic review

**DOI:** 10.1186/s12957-021-02306-2

**Published:** 2021-06-29

**Authors:** Qianyun Pang, Liping Duan, Yan Jiang, Hongliang Liu

**Affiliations:** Department of Anesthesiology, Chongqing University Cancer Hospital, Hanyu Road No. 181, Shapingba District, Chongqing, 400030 China

**Keywords:** Enhanced recovery after surgery, Cancer surgery, Long-term outcome, Oncologic outcome

## Abstract

**Background:**

Clinical evidence has proved that enhanced recovery after surgery (ERAS) can improve short-term clinical outcomes after various types of surgeries, but the long-term benefits have not yet been examined, especially with respect to cancer surgeries. Therefore, a systematic review of the current evidence was conducted.

**Methods:**

The Pubmed, Cochrane Library, Embase, and Web of Science databases were searched using the following key words as search terms: “ERAS” or “enhanced recovery” or “fast track”, “oncologic outcome”, “recurrence”, “metastasis”, “long-term outcomes”, “survival”, and “cancer surgery”. The articles were screened using the inclusion and exclusion criteria, and the data from the included studies were extracted and analyzed.

**Results:**

A total of twenty-six articles were included in this review. Eighteen articles compared ERAS and conventional care, of which, 12 studies reported long-term overall survival (OS), and only 4 found the improvement by ERAS. Four studies reported disease-free survival (DFS), and only 1 found the improvement by ERAS. Five studies reported the outcomes of return to intended oncologic treatment after surgery (RIOT), and 4 found improvements in the ERAS group. Seven studies compared high adherence to ERAS with low adherence, of which, 6 reported the long-term OS, and 3 showed improvements by high adherence. One study reported high adherence could reduce the interval from surgery to RIOT. Four studies reported the effect of altering one single item within the ERAS protocol, but the results of 2 studies were controversial regarding the long-term OS between laparoscopic and open surgery, and 1 study showed improvements in OS with restrictive fluid therapy.

**Conclusions:**

The use of ERAS in cancer surgeries can improve the on-time initiation and completion of adjuvant chemotherapy after surgery, and the high adherence to ERAS can lead to better outcomes than low adherence. Based on the current evidence, it is difficult to determine whether the ERAS protocol is associated with long-term overall survival or cancer-specific survival.

**Supplementary Information:**

The online version contains supplementary material available at 10.1186/s12957-021-02306-2.

## Background

The concept of enhanced recovery after surgery (ERAS) regimen was first introduced by Kehlet in 1997 [1]; it was then gradually accepted and widely used in nearly all types of surgeries. Clinical evidence has proved that ERAS not only improves clinical outcomes and quality of care, but also significantly reduces the cost of hospitalization [2–6]. However, the majority of the clinical evidence regarding the benefits of ERAS describes short-term outcomes; the long-term benefits of ERAS are not fully elucidated, especially with respect to cancer surgeries.

Due to the increasing number of cancer cases, the proportion of cancer surgeries among all surgeries is increasing worldwide. Patients undergoing cancer surgeries commonly need neoadjuvant chemotherapy afterwards, and the on-time initiation and completion of chemotherapy after surgery are critical for the prognosis [7], as recurrence and metastasis can directly influence quality of life and long-term survival. The short-term benefits of ERAS are postulated to be associated with its long-term benefits [8], but this has not been fully verified for cancer surgeries. Thus, we conducted a literature search to identify studies on oncologic and long-term outcomes that examined: (1) ERAS versus conventional care, (2) high adherence to ERAS versus low adherence, and (3) the effects of altering one single item within the ERAS protocol.

## Methods

We construct the framework of this systematic review in accordance with the 2009 PRISMA (Preferred Reporting Items for Systematic Reviews and Meta-Analyses) guidelines [9]. The PRISMA checklist is presented in Additional file 1. The protocol of this systematic review was registered in INPLASY with the registration number INPLASY202150099, and the link https://inplasy.com/inplasy-2021-5-0099/.

### Literature search

The Pubmed, Cochrane Library, Embase, and Web of Science databases were searched from Jan 2000 to April 2021. The key terms were “ERAS” or “enhanced recovery” or “fast track”, “oncologic outcome”, “recurrence”, “metastasis”, “long-term outcomes”, “survival”, “cancer surgery”, and various combinations of these key terms were used. The detailed search strategy in Pubmed was shown as followed:

#1 “Enhanced Recovery After Surgery”[Mesh]

#2 ((Enhanced Recovery After Surgery) OR (ERAS)) OR (fast track)

#3 Cancer Surgery

#4 (#1 OR #2) AND #3

#5 ((((((oncologic outcome) OR (recurrence)) OR (metastasis)) OR (long-term outcomes)) OR (survival)) OR (cancer specific death)) OR (oncologic)

#6 #4 AND #5

The detailed search strategies in other databases were presented in Additional file 2. The reference lists of the included studies were checked for potentially eligible articles. The languages of the full-text articles were restricted to English and Chinese.

### Inclusion and exclusion criteria

The inclusion criteria included studies comparing ERAS and conventional care, comparing different levels of adherence to ERAS, examining alterations of one single item within the ERAS protocol, studies with adult patients (> 18 years old) undergoing cancer surgery, studies describing oncologic outcomes (return to intended oncologic treatment after surgery (RIOT), recurrence, metastasis, and cancer-specific survival) or long-term outcomes (overall survival and quality of life), and prospective or retrospective studies. Exclusion criteria included studies of pediatric surgery, studies describing only short-term outcomes, studies without full text, review articles, or case reports.

### Data extraction

Two reviewers (QP and LD) independently extracted data from all included articles, which included the author, publication time, study design, patient age, major diagnosis, surgical type, patient groups and sample size, key elements of the ERAS protocol, oncologic and long-term outcomes, and findings. Disagreements were resolved through consensus between reviewers; if necessary, an additional reviewer was consulted to resolve the dispute.

### Data analysis

The methodological quality was evaluated by the Newcastle-Ottawa Scale (NOS) for cohort studies and by the Jadad score for RCTs. The highest NOS score was of 9 stars and the highest Jadad score was 7. Information on major outcomes of interest was recorded, including oncologic outcomes (RIOT, recurrence, metastasis, and cancer-specific survival), long-term overall survival, and quality of life.

## Results

### Literature search and retrieval

**Fig. 1:**
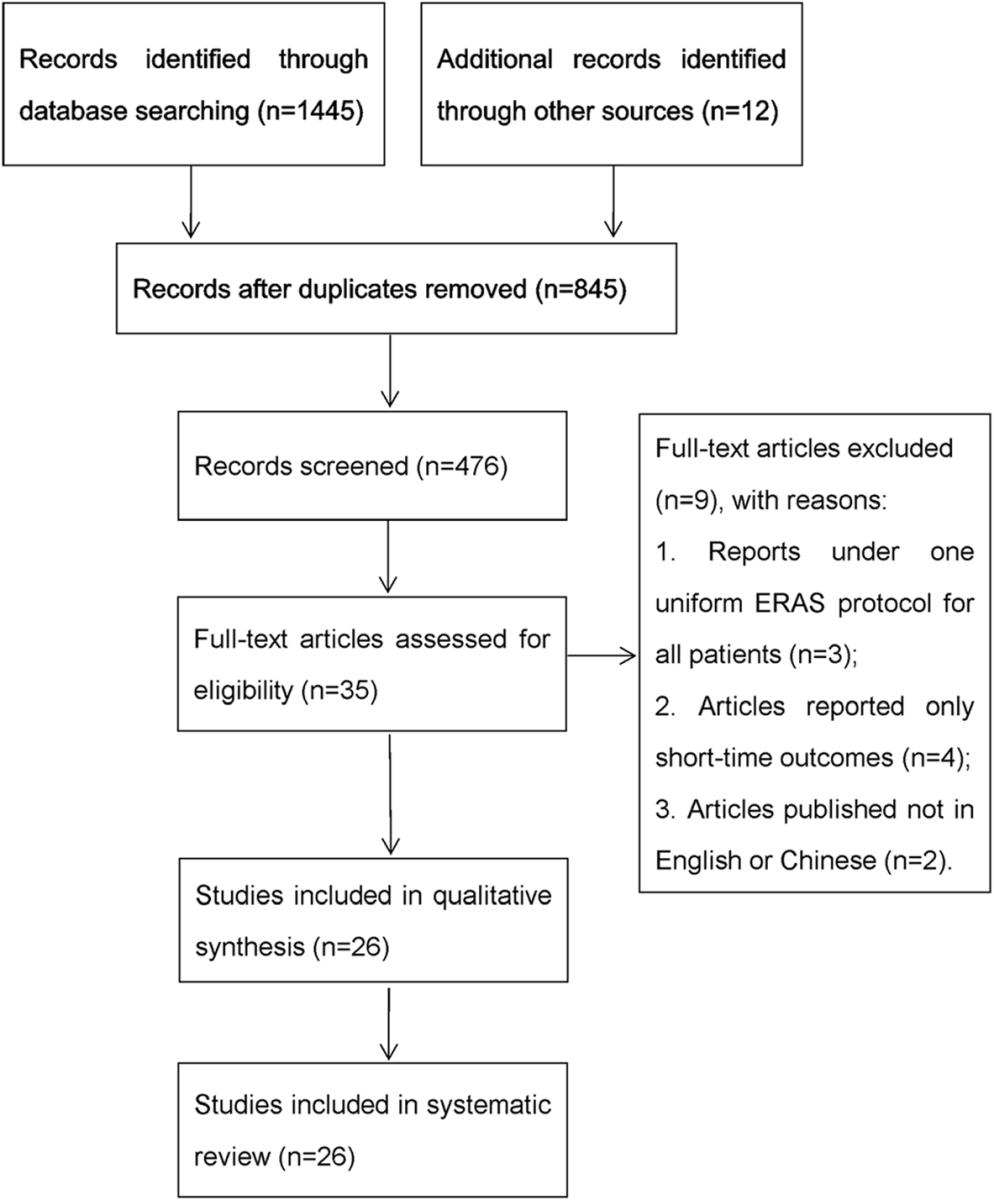
PRISMA flowchart of systematic strategy

A total of 845 relevant publications were identified through the keywords. The full versions of 35 articles were retrieved after screening and conducting a detailed selection process, and 26 articles [10–35] eventually met the inclusion criteria and underwent data extraction. The details of the screening process are presented in Fig. 1.

### Study characteristics

The characteristics and quality of the included articles are presented in Table 1. Of these 26 articles, 3 were randomized trials [12, 13, 15], which were evaluated by Jadad score, and the other 23 were prospective or retrospective cohort studies, which were evaluated by the NOS. Eighteen articles compared conventional care with ERAS [10–21, 29–34], 7 studies compared high adherence to ERAS with low adherence [16, 22–26, 34], and 4 studies [27–29, 35] investigated the outcome of one single item altered within ERAS protocol. The elements of the ERAS protocol in each article are presented in Table 2. The number of ERAS elements in the included articles ranged from 3 to 18, and 17 articles described a protocol containing more than 10 elements [11, 13–18, 21, 22, 24, 26, 27, 29, 30, 32–34].

**Table 1:** Characteristics of included studies

Author	Year	Study design	Surgery	Groups	Patient age (year)	Outcomes	NOS/Jadad score*
**Non-ERAS vs ERAS**							
Oakley et al. [10]	2016	Retrospective cohort	Esophagi-gastric resection	Non-ERAS (n = 81) ERAS (n = 66)	Median 78.8 Median 78.5	OS	8
Pang et al. [11]	2018	Prospective cohort	Radical cystectomy	Non-ERAS (n = 393) ERAS (n = 60)	Median 66 Median 71	OS CSS	7
Ziegelmueller et al. [12]	2019	Prospective randomized	Radical cystectomy	Non-ERAS (n = 38) ERAS (n = 60)	Median 67.5 Median 70	OS CSS	4*
Ye [13]	2017	Prospective randomized	Radical gastrectomy	Non-ERAS (n = 38) ERAS (n = 47)	Mean 43.2 Mean 45.2	OS	4*
Yang et al. [14]	2020	Retrospective cohort	Radical gastrectomy	Non-ERAS (n = 521) ERAS (n = 521)	Mean 63	OS	8
Liu et al. [15]	2020	Randomized control trial	Craniotomy	Non-ERAS (n = 29) ERAS (n = 36)	18–65	Life quality	6*
Passeri et al. [16]	2020	Prospective cohort	Pancreatico-duodenectomy	Non-ERAS (n = 86) ERAS (n = 86)	Median 67	OS DFS	6
Day et al. [17]	2015	Prospective cohort	Hepatectomy	Non-ERAS (n = 43) ERAS (n = 75)	Median 60 Median 59	Failure to RIOT Days to RIOT	8
Lohsiriwat [18]	2014	Prospective cohort	Colorectal cancer resection	Non-ERAS (n = 40) ERAS (n = 20)	Mean 62 Mean 57.6	Days to RIOT	7
Hassinger et al. [19]	2019	Retrospective cohort	Colorectal cancer resection	Non-ERAS (n = 174) ERAS (n = 189)	Median 61.1 Median 62.4	Rate of on time to RIOT	8
Nelson et al. [20]	2019	Prospective cohort	Lung resection	Non-ERAS (n = 230) ERAS (n = 92)	Median 66	Days to RIOT Rate of completing RIOT	7
Li et al. [21]	2020	Prospective cohort	Pancreatico-duodenectomy	Non-ERAS (n = 141) ERAS (n = 203)	Median 58.2 Median 58.9	Days to RIOT	7
Wang et al. [29]	2021	Retrospective cohort	Colorectal cancer resection	Non-ERAS (1) (n = 142) ERAS (1) (n = 125) Non-ERAS (2) (n = 138) ERAS (2) (n = 137)	Median 57.65 Median 56.22 Median 58.45 Median 57.18	OS; DFS	7
Zhang et al. [30]	2021	Retrospective cohort	Hepatectomy	Non-ERAS (n = 463) ERAS (n = 463)	Median 62.8 Median 63.2	OS; DFS	7
Quiram et al. [31]	2019	Retrospective cohort	Rectal cancer resection	Non-ERAS (n = 280) ERAS (n = 320)	Median 58.9 Median 58.2	OS; DFS; local recurrence; metastasis	7
Zhu et al. [32]	2019	Retrospective cohort	Pancreatico-duodenectomy	Non-ERAS (n = 69) ERAS (n = 64)	Median 64.1 Median 64.3	OS	6
Tian et al. [33]	2020	Retrospective cohort	Gastrectomy	Non-ERAS (n = 365) ERAS (n = 365)	Median 59.4 Median 59.5	OS; CSS	7
Lohsiriwat et al. [34]	2021	Prospective cohort	Colorectal cancer resection	Non-ERAS (n = 279) ERAS (n = 70)	Mean 63.1 Mean 62.1	OS	7
**High adherence vs low adherence to ERAS**							
Passeri et al. [16]	2020	Prospective cohort	Pancreatico-duodenectomy	High (n = 42) Low (n = 44)	Median 67	OS; DFS	6
Viannay et al. [22]	2019	Retrospective cohort	Colectomy	High (n = 52) Low (n = 154)	Mean 68.2 Mean 71.8	OS; survival of metastasis	7
Gustafsson et al. [23]	2016	Retrospective cohort	Colorectal resection	High (n = 273) Low (n = 638)	Mean 68.7 Mean 69.6	Local recurrence; OS; CSS	7
Pisarska et al. [24]	2019	Prospective cohort	Colorectal resection	High (n = 241) Low (n = 109)	Mean 63.8 Mean 64.9	OS	7
St-Amour et al. [25]	2020	Retrospective cohort	Surgery for liver and pancreatic cancer	High (n = 42) Low (n = 91)	Median 67	Days to RIOT in young patients; DFS in young patients	6
Rubinkiewicz et al. [26]	2020	Retrospective cohort	Gastrectomy	High (n = 34) Low (n = 44)	Mean 61.1 Mean 61.3	OS	7
Lohsiriwat et al. [34]	2021	Prospective cohort	Colorectal cancer resection	High (n = 232) Low (n = 88)	Mean 62.5 Mean 65.1	OS	7
**Alteration of single item within ERAS protocol**							
Curtis et al. [27]	2018	Prospective cohort	Colorectal resection	Laparoscopy surgery (n = 383) Open surgery (n = 373)	Unclear	OS	7
Wang et al. [29]	2021	Retrospective cohort	Colorectal cancer resection	Laparoscopy (n = 125) Open surgery (n = 137)	Mean 56.2 Mean 57.1	OS; DFS	7
Asklid et al. [28]	2017	Prospective cohort	Colorectal resection	Restrictive fluid therapy (n = 145) Liberal fluid therapy (n = 753)	Mean 69.3	Local recurrence; OD; CSD	7
Kato et al. [35]	2021	Retrospective cohort	Rectosigmoid resection	Early oral feeding (n = 106) Conventional oral feeding (n = 95)	Median 60 Median 62	Days to RIOT	6

*RIOT* return to intended oncologic therapy, *OS* overall survival, *CSS* cancer-specific survival, *DFS* disease-free survival, *OD* overall death, *CSD* cancer-specific death

*Study evaluated by Jadad score

**Table 2:** ERAS protocol elements in the included studies

References No.	10	11	12	13	14	15	16	17	18	19	20	21	22	23	24	25	26	27	28	29	30	31	32	33	34	35
Education and counseling	-	+	-	+	+	+	+	+	+	-	+	+	+	+	+	+	+	+	+	+	+	+	+	+	+	-
Bowel preparation	-	+	+	+	-	+	+	+	-	+	-	+	+	+	+	+	-	+	+	+	+	-	-	-	+	-
Carbohydrate loading	-	+	+	+	+	+	+	+	-	+	+	+	+	+	+	+	+	+	+	-	+	-	-	-	+	+
Regional anesthesia	+	+	-	+	+	+	+	+	+	-	+	+	-	+	-	-	+	+	+	+	-	+	+	+	-	-
Steroids	-	-	-	-	-	-	-	+	-	-	+	-	+	-	-	-	-	-	-	-	+	-	-	-	-	-
Body temperature	-	+	-	+	+	+	+	-	+	-	-	+	+	+	-	-	-	-	+	+	+	-	+	+	+	-
Fluid therapy	-	+	-	+	+	+	+	+	-	+	+	-	+	-	+	-	+	+	-	+	+	+	+	+	+	-
Micro-invasive surgery	-	+	-	-	+	+	+	-	-	-	-	-	+	-	+	-	+	+	-	+	+	+	-	-	+	-
Multimodal analgesia	+	+	+	+	+	+	+	+	+	+	+	+	+	-	+	+	+	+	-	+	+	+	+	+	+	-
NG tube	+	+	+	+	+	-	+	+	+	-	-	+	+	-	+	-	+	+	-	+	+	-	+	+	+	-
Drainage	+	+	+	-	+	-	+	-	+	-	-	+	+	-	+	+	+	+	-	+	+	-	+	+	+	-
Urinary catheter	-	+	-	+	+	-	+	-	+	-	-	-	+	-	+	-	+	-	-	+	-	+	+	+	+	-
Prophylactic antithrombosis	-	+	-	-	+	+	+	-	-	-	-	-	+	-	+	+	+	-	-	-	+	-	+	+	+	-
Prophylactic antibiotics	-	+	-	-	-	-	+	-	-	-	+	+	-	-	+	+	+	-	-	-	+	-	+	-	+	+
Prophylactic anti-emetics	-	+	-	-	-	+	+	-	+	-	-	-	+	-	+	-	-	-	-	-	+	-	-	-	+	-
Early oral feeding	+	+	-	+	+	+	+	+	+	+	+	+	+	-	+	-	+	+	+	+	-	+	+	+	+	+
Early mobilization	+	+	+	+	+	+	+	+	+	+	+	+	+	+	+	-	+	+	+	+	+	+	+	+	+	-
Discharge planning	-	-	-	+	-	-	-	+	+	-	-	-	-	-	-	-	-	-	-	-	-	-	-	-	-	-
Others	-	+	-	-	-	-	+	-	-	-	-	-	+	+	-	+	-	-	+	+	+	-	+	+	+	+
Elements (n)	6	17	6	12	13	12	17	11	11	6	9	11	16	7	14	8	13	11	8	13	15	8	13	12	16	3

“+”, element explicitly listed in ERAS protocol; “-”, element not explicitly listed in ERAS protocol

### Outcomes and findings

The outcomes and findings of the included studies are displayed in Table 3. Seventeen studies described oncologic outcomes [11, 12, 16–23, 25, 28–31, 33, 35], and 20 studies described long-term outcomes [10–16, 22–34].

**Table 3:** Effects of ERAS on oncologic and long-term outcomes

Outcomes			Study	*P* value	Findings	Follow-up time
Non-ERAS vs ERAS	OS		Pang et al. [11]	*P* = 0.9	NS	10 years
			Ziegelmueller et al. [12]	*P* = 0.550	NS	7 years
			Oakley et al. [10]	*P* = 0.57	NS	5 years
			Yang et al. [14]	*P* = 0.007	ERAS improved OS	5 years
			Ye et al. [13]	*P* = 0.066	NS	3 years
			Passeri et al. [16]	*P* = 0.72	NS	2 years
			Wang et al. [29]	*/*	NS	10–20 months
			Zhang et al. [30]	*P = 0.035*	ERAS improved OS	3 years
			Quiram et al. [31]	*P = 0.464*	NS	5 years
			Zhu et al. [32]	*P = 0.810*	NS	1 year
			Tian et al. [33]	*P = 0.013*	ERAS improved OS	5 years
			Lohsiriwat et al. [34]	*P = 0.014*	ERAS improved OS	5 years
	CSS		Pang et al. [11]	*P* = 0.9	NS	10 years
			Ziegelmueller et al. [12]	*P* = 0.725	NS	7 years
			Tian et al. [33]	*P = 0.033*	ERAS improved CSS	5 years
	DFS		Passeri et al. [16]	*P* = 0.38	NS	2 years
			Wang et al. [29]	*/*	NS	10–20 months
			Zhang et al. [30]	*P = 0.007*	ERAS improved DFS	3 years
			Quiram et al. [31]	*P = 0.272*	NS	5 years
	Recurrence		Quiram et al. [31]	*P = 0.157*	NS	3 years
	Metastasis		Quiram et al. [31]	*P = 0.129*	NS	5 years
	Life quality	Physical functioning	Liu et al. [15]	*P* = 0.038	ERAS improved life quality	6 months
		Nausea/vomiting		*P* = 0.048		
		Motor dysfunction		*P* = 0.019		
	RIOT	Failure to RIOT	Day et al. [17]	*P* = 0.373	NS	/
		Days to RIOT	Day et al. [17]	*P* = 0.134	NS	
			Lohsiriwat [18]	*P* = 0.009	ERAS reduced interval to RIOT	
			Nelson et al. [20]	*P* = 0.364	NS	
			Li et al. [21]	*P* = 0.000	ERAS reduced interval to RIOT	
		Rate of on time to RIOT	Hassinger et al. [19]	*P* = 0.022	ERAS improved the on-time initiation of RIOT	
		Rate of completing RIOT	Nelson et al. [20]	*P* < 0.001	ERAS improved RIOT completion	
High adherence vs low adherence	OS		Gustafsson et al. [23]	*P* < 0.001	High adherence improved OS	5 years
			Lohsiriwat et al. [34]	*P = 0.007*	High adherence improved OS	5 years
			Viannay et al. [22]	*P* = 0.632	NS	3 years
			Pisarska et al. [24]	*P* = 0.0007	High adherence improved OS	
			Rubinkiewicz et al. [26]	*P* = 0.75	NS	
			Passeri et al. [16]	*P* = 0.14	NS	2 years
	CSS		Gustafsson et al. [23]	*P* = 0.020	High adherence improved CSS	5 years
	DFS		St-Amour et al. [25]	*P* = 0.000	High adherence improved DFS	3 years
			Passeri et al. [16]	*P* = 0.81	NS	2 years
	Survival of metastasis		Viannay et al. [22]	*P* = 0.668	NS	3 years
	Local recurrence		Gustafsson et al. [23]	*P* = 0.211	NS	5 years
	Days to RIOT in young patients		St-Amour et al. [25]	*P* = 0.001	High adherence reduced interval to RIOT	3 years
Alteration of single item within ERAS protocol	OS		Curtis et al. [27]	*P* = 0.009	Laparoscopy surgery improved OS	5 years
			Wang et al. [29]	*/*	NS	10–20 months
	DFS		Wang et al. [29]	*/*	NS	10–20 months
	Local recurrence		Asklid et al. [28]	*P* = 0.981	NS	5 years
	OD			*P* = 0.006	Restrictive fluid therapy improved survival	
	CSD			*P* = 0.008		
	Days to RIOT		Kato et al. [35]	*P = 0.08*	NS	/

*OS* overall survival, *CSS* cancer-specific survival, *DFS* disease-free survival, *RIOT* return to intended oncologic therapy, *OD* overall death, *CSD* cancer-specific death, *NS* no significance

#### Non-ERAS vs ERAS

Twelve studies [10–14, 16, 29–34] reported long-term overall survival (OS), and the follow-up time ranged from 10 months to 10 years. Four studies [14, 31, 33, 34] showed ERAS was associated with increasing long-term OS, while the others found no differences between conventional care and ERAS. One study [15] reported quality of life, and the results showed that ERAS improved 6-month quality of life after surgery.

Three studies reported long-term cancer-specific survival (CSS) [11, 12, 33], and 4 studies reported long-term disease-free survival (DFS) [16, 29–31].The follow-up time ranged from 10 months to 10 years, 1 study showed that ERAS could improve CSS [33] and DFS [30], while the others found no differences. One study [31] reported recurrence and metastasis, the result found no difference between conventional care and ERAS.

Five studies [17–21] reported the outcomes of RIOT; 2 out of the 5 studies showed a reduced interval from surgery to RIOT with the ERAS group [18, 21], 1 showed improvement in RIOT completion with ERAS [20], 1 showed an improvement in the rate of on time to RIOT with ERAS [19], and 1 showed no differences in the completion of RIOT and the interval from surgery to RIOT between non-ERAS and ERAS groups [17].

#### High adherence to ERAS vs low adherence to ERAS

Seven studies compared high adherence to ERAS and low adherence to ERAS [16, 22–26, 34]. Long-term OS was reported in six studies, and the follow-up time ranged from 2 to 5 years. Half of the studies showed no differences in OS between high and low adherence [16, 22, 26], and half showed improvements in OS by high adherence to ERAS [23, 24, 34]. Two studies reported DFS [16, 25]; one showed that high adherence to ERAS could improve 3-year DFS [25], and the other did not find any difference [16]. One study showed that high adherence to ERAS could improve 5-year CSS [23], 2 studies reported that high adherence to ERAS had no effect on 3-year metastasis or 5-year recurrence [22, 23], and 1 study showed that high adherence to ERAS reduced the interval from surgery to RIOT [25].

#### Alteration of one single item within ERAS protocol

Four studies reported the effect of altering one single item within the ERAS protocol on long-term survival and local recurrence [27–29, 35]. The follow-up time ranged from 10 months to 5 years. Two studies compared laparoscopic with open surgery within the ERAS protocol, of which, 1 study revealed improvements in OS with laparoscopic surgery [27], and the other did not find any difference [29]. One study compared restrictive with liberal fluid therapy within the ERAS protocol, and revealed improvements in OS with restrictive fluid therapy, but no differences were found in local recurrence [28]. One study compared early oral feeding with conventional oral feeding within ERAS protocol, and found no difference in days to RIOT after surgery [35].

## Discussion

Since the concept of ERAS was proposed more than 20 years ago, these regimen has been widely applied in cardiac surgery, general surgery, neurosurgery, head and neck surgery, thoracic surgery, gynecologic surgery, urinary surgery, and orthopedic surgery. Clinical evidence has proved that ERAS can improve short-term postoperative outcomes in both cancer and non-cancer surgeries. For cancer patients, quality of life and long-term survival are the most important factor. However, whether the short-term benefits of ERAS are associated with long-term benefits in patients undergoing cancer surgeries has not yet been verified.

The results of this review showed that ERAS improved the on-time initiation and completion of RIOT after cancer surgeries. Various factors are considered when deciding to start adjuvant chemotherapy after surgery, of which, the patient’s condition is the most important one [36, 37]. ERAS improves short-term outcomes including patient conditions, so cancer patients undergoing ERAS can receive on-time and higher-rate chemotherapy after surgery. The interval from surgery to adjuvant chemotherapy is commonly 30 to 60 days; the interval in the included studies ranged from 33 days to 68 days, so the initiation or completion of RIOT is a mid-term outcome measure [38], not a short-term or long-term measure. This review showed that ERAS could not improve long-term OS or CSS in the majority of the included studies. Theoretically, improved short-term outcomes are postulated to be associated with long-term outcomes; however, the results of this review suggested that ERAS-induced improvement of mid-term oncologic outcomes was not associated with long-term survival, and no studies have compared the effects of ERAS and non-ERAS on local recurrence and metastasis until now. It was reported that the long-term prognosis after bladder cancer surgery was determined by tumor stage, presence of metastasis at surgery, and resection status [12]. ERAS might not play the pivotal role in long-term prognosis.

Adherence to ERAS is critical for short-term outcomes. Higher adherence is associated with better short-term outcomes [39, 40]. This review included 6 studies comparing long-term survival between high adherence to REAS and low adherence to ERAS [16, 22–24, 34], and half reported improvements in survival by high adherence, and half did not. One study reported improvements in on-time initiation of RIOT with high adherence [25]. Till now, the criteria of the adherence to ERAS have not been well defined, and the level of high adherence ranged from 67 to 85% in the included 7 studies. Higher adherence is more difficult to implement, and 70% adherence to ERAS is considered a common standard of high adherence and an achievable target in the clinic [40].

Within the ERAS protocol, restrictive fluid therapy could reduce 5-year OD and CSD in one study, while the effects on long-term survival were controversial between laparoscopic and open surgery in two studies, days to RIOT was not improved by early oral feeding compared with conventional oral feeding. Till now, studies on alteration of one single item within ERAS protocol were scarce. Furthermore, the ERAS protocol is a combination therapy, and the pre-, intra-, and postoperative items are combined together to improve postoperative outcomes. So alteration of one single item within ERAS protocol might not be so important for the oncologic and long-term outcomes. In the 26 included studies in this review, the most frequently used components of ERAS included patient education and counseling, avoidance of bowel preparation, carbohydrate loading, regional anesthesia, targeted fluid therapy, multimodal analgesia, early removal of nasogastric tubes and drainage, early oral feeding, and mobilization. However, the number of ERAS components ranged from 3 to 18 in these studies, and different numbers of ERAS components might cause different outcomes. Perioperative steroids or discharge planning were used in few studies. Therefore, the standard ERAS protocol should be used for each type of cancer surgery in accordance with the ERAS protocols in the ERAS interactive audit system, so that the oncologic and long-term outcomes under a defined framework can be fully assessed.

There are some limitations in this systematic review. First, tumor entities and surgical procedures were quite different; although in each study the results were comparable between groups, the results between studies were not comparable. Second, ERAS protocols and the follow-up time in these included trials were not uniform, so that meta-analysis could not be conducted. Third, DFS is a most important endpoint in oncologic studies, but only few studies reported the effect of ERAS on long-term DFS; regardless of the fact that ERAS could improve RIOT after surgery, the long-term oncologic outcomes after ERAS were still unclear. Fourth, the majority of the included studies were of cohort studies, and large RCTs are needed to verify the effects of the ERAS protocol on oncologic and long-term outcomes in the future.

## Conclusions

This systematic review identified 26 studies with variable patient populations, cancer surgeries, and ERAS protocol implementation. Our results showed that ERAS protocols in cancer surgeries can improve the on-time initiation and completion of adjuvant chemotherapy after surgery, and high adherence to ERAS lead to better outcomes than low adherence. Based on the current evidence, it is difficult to determine whether the ERAS protocol is associated with recurrence or metastasis and long-term survival. Future efforts should be directed towards the application of a standard ERAS protocol in each type of cancer surgery and evaluation of its impact using larger, comparative multi-center studies at high- and low-volume medical centers.

## Supplementary Information

**Additional file 1.** PRISMA 2009 Checklist.

**Additional file 2.** Search strategies for other database.

## Data Availability

The data-sets supporting the conclusion are included in the article.

## Abbreviations

ERAS: Enhanced recovery after surgery
OS: Overall survival
CSS: Cancer-specific survival
DFS: Disease-free survival
RIOT: Return to intended oncologic therapy
