# Supplementary material for: Oncologic and long-term outcomes of enhanced recovery after surgery in cancer surgeries — a systematic review
Source: World J Surg Oncol. 2021 Jun 29;19:191. doi: 10.1186/s12957-021-02306-2 (PMC8243430; doi:10.1186/s12957-021-02306-2)
Supplement: Supplementary file 2 — Additional file 2. Search strategies for other database. [file 12957_2021_2306_MOESM2_ESM.doc]

**Cochrane Library:**

PICO search^BETA^:

Surgery (Population)

AND Perioperative Enhanced Recovery Program (Intervention)

**Embase：**

PICO:

Cancer surgery (Population)

AND Enhanced recovery after surgery (Intervention)

**Web of Science**

#1 “Enhanced recovery after surgery"[Mesh]

#2 “Cancer surgery”

#3 #1 AND #2
